# Editorial: One volume down, more to come

**DOI:** 10.1120/jacmp.v9i4.2964

**Published:** 2008-10-29

The publication of this issue of the Journal of Applied Clinical Medical Physics marks the conclusion of my first year as Editor‐in‐Chief. The experience of editing a scientific journal has been a rewarding one for me, placing me in contact with many of my clinical and scientific colleagues in a collaborative venture that, we hope, contributes to the quality of medical physics practice throughout the world. I have interacted in previous years with the JACMP as an author. Finding myself on the other side of the fence has given me a greater appreciation of the role of an editor and, in particular, a greater appreciation for the fine contributions my predecessors in the position as Editor‐in‐Chief have made to our profession of medical physics.

I am pleased that the JACMP has established itself in, what is to the best of my knowledge, a unique role as an open access, online journal in the medical physics community. There is no need to repeat our journal's philosophy regarding open access; my predecessors have articulated this philosophy very well. Open access appears to be the wave of the future. The National Institutes of Health, for example, has mandated that

“…all investigators funded by the NIH submit or have submitted for them to the National Library of Medicine's PubMed Central an electronic version of their final, peer‐reviewed manuscripts upon acceptance for publication…”^(1)^

More recently, on February 12 of this year, Harvard University's Faculty of Arts and Sciences adopted a policy that requires that scholarly articles by faculty members be available free to the public online.^(2)^

As of October 1 of this year, over 3500 journals are listed in the Directory of Open Access Journals.^(3)^ Three or four other journals that address topics in medical physics are listed in this directory, but the JACMP is, at least at present, the only one specifically dedicated to the profession of medical physics. Whether or not this will continue to be true in the future is anyone's guess. On one hand, we would welcome other medical physics journals to join the open access community but, on the other hand, were that to happen, the JACMP would lose some of its uniqueness.

Now that my first year as Editor‐in‐Chief is coming to a close, I would like to share with you a few statistics about the JACMP, some of which are presented in Table 1.

**Table 1:** Some JACMP Publication Statistics

*Yr/Qtr*	*# submissions*	*# accepted*	*# declined*	*% declined*	*Mean time to completion of first review*
2007/3	31	18	8	26%	25 days
2007/4	34	18	13	38%	25 days
2008/1	33	8	16	49%	29 days
2008/2	44	9	15	34%	24 days
2008/3	33	2	3	9%	—

It should be noted that the number of acceptances plus the number of rejections does not equal the number of submissions, as papers are sometimes withdrawn (as is the case with 3 papers from the 3rd quarter of 2007), and many papers, especially recent submissions, are still in review. The number of submissions appears to be steady at around 33 per quarter, although we saw a substantial increase during the second quarter of 2008. It appears that approximately 1/3 of submissions are declined; the low value for the 3rd quarter of 2008 is simply a result of the fact that most manuscripts submitted during that period are still under review. A rejection rate of approximately 1/3 is a rate that I am presently comfortable with, although increases in the number of submissions may result in a change in the bar. The mean time to complete the first round of review appears to be steady at around 25 days, which is also, I believe, a good figure, attesting to the diligence of our Associate Editors and Reviewers.

A journal such as the JACMP would not exist without the cooperation of our Associate Editors and Reviewers. These are the heroes who are charged with reading the manuscripts and critically assessing their suitability for the JACMP. Associate Editors are listed below:

Associate Editors (including Guest Associate Editors): Nzhde Agazaryan, Salahuddin Ahmad, John Antolak, Peter Balter, Pat Cadman, Marco Carlone, Nathan Childress, Geoff Clarke, Laurence Court, Jianrong Dai, Larry DeWerd, Gino Fallone, John Gibbons, Michael Gossman, Stephen Kry, Eduardo Moros, Firas Mourtada, Ben Nelms, Niko Papanikolaou, Bill Pavlicek, Matt Podgorsak, Chet Ramsey, Jim Rodgers, John Rong, Bill Salter, Mehrdad Sarfaraz, Tim Solberg, Frank Van Den Heuvel, Sastry Vedam, Lu Wang, Chuck Willis, Al Zacarias, Ron Zhu

Over 200 individuals served as Reviewers, and they are listed in a Supplementary File. Please refer to this file to acknowledge their immense contribution to the JACMP. Thanks, of course, to the many scientists and clinicians who submitted manuscripts. Without a continuous stream of good manuscripts, our Associate Editors and Reviewers would have nothing to do. And, finally, I would be remiss if I did not thank you, the readers of this journal. Without a readership, we would have no journal. Thank you for your continued support. Please join me in looking forward to our Tenth Anniversary Volume.

George Starkschall, PhD

Editor‐in‐Chief

November 15, 2008
